# Microbiota-gut-brain axis in cerebral palsy: from mechanisms to interventions

**DOI:** 10.3389/fmed.2026.1911417

**Published:** 2026-07-30

**Authors:** Fei Xie, Chao Bai, Jian Xu, Xinping Luan

**Affiliations:** 1 Department of Neurosurgery, The Seventh Affiliated Hospital of Xinjiang Medical University, Urumqi, Xinjiang, China; 2 Cerebral Palsy Center in Neurosurgery, Second Affiliated Hospital of Xinjiang Medical University, Urumqi, Xinjiang, China

**Keywords:** cerebral palsy, constipation, gut microbiota, microbiota-gut-brain axis, neuroinflammation, nutritional intervention, probiotics

## Abstract

Cerebral palsy (CP) is the most common cause of chronic motor disability in childhood and arises from non-progressive injury to the developing brain. Despite the static nature of the primary lesion, children with CP frequently experience evolving gastrointestinal, nutritional, inflammatory, sleep, cognitive, and seizure-related comorbidities that substantially influence functional recovery and caregiver burden. The microbiota-gut-brain axis (MGBA) provides a biologically plausible framework linking these multisystem manifestations. Emerging evidence suggests that children with CP may exhibit reduced gut microbial diversity, depletion of short-chain fatty acid (SCFA)-producing taxa, enrichment of opportunistic bacteria, and microbiome remodeling related to diet, constipation, antiepileptic drug exposure, oral inflammation, and care patterns. Dysbiosis may interact with CP through epithelial barrier disruption, lipopolysaccharide translocation, systemic immune activation, altered tryptophan-kynurenine metabolism, abnormal bile acid and SCFA signaling, vagal and enteric nervous system pathways, hypothalamic-pituitary-adrenal axis dysregulation, and microglial priming. These mechanisms may create a self-reinforcing cycle in which early brain injury promotes gut dysfunction, gut dysfunction reshapes the microbiome, and dysbiotic immune-metabolic signals further amplify symptom burden. Current interventions, including nutritional optimization, constipation protocols, dietary fiber, probiotics, prebiotics, synbiotics, oral health management, and family-centered care, show promise for improving bowel symptoms and selected microbial or inflammatory indices. However, most clinical studies remain small, short-term, and focused on constipation rather than long-term neurodevelopmental outcomes. All CP-specific human evidence currently demonstrates association rather than causation; no study has established that dysbiosis initiates CP or causally drives its neurological phenotype. Accordingly, mechanistic pathways are presented as testable hypotheses, not validated causal mechanisms in CP. This review synthesizes current evidence on the MGBA in CP, outlines mechanistic pathways, evaluates therapeutic opportunities, and proposes future directions for biomarker-driven and stratified intervention trials.

## 1 Introduction

Cerebral palsy (CP) comprises a heterogeneous group of permanent disorders of movement and posture caused by disturbances in the developing fetal or infant brain. The causative brain injury is classically described as non-progressive; however, the clinical phenotype is dynamic across childhood and adolescence. Feeding difficulties, dysphagia, gastroesophageal reflux, delayed gastric emptying, constipation, malnutrition, epilepsy, sleep disturbance, pain, and cognitive or behavioral impairment commonly coexist with the motor disorder and can substantially shape rehabilitation potential, quality of life, and family burden (1–3). Accordingly, CP should not be conceptualized only as a disorder of descending motor pathways, but rather as a chronic neurodevelopmental condition in which neural injury, immunity, metabolism, nutrition, and environment interact over time (3–5).

The microbiota-gut-brain axis (MGBA) is a bidirectional communication network involving the intestinal microbiota, epithelial and blood-brain barriers, mucosal immunity, microbial metabolites, enteric and autonomic neural circuits, neuroendocrine signaling, and the central nervous system. Experimental and translational studies indicate that the gut microbiome contributes to brain development, synaptic refinement, myelination, microglial maturation, stress responsivity, and behavioral regulation (6–8). This concept is highly relevant to CP because many children with CP live with persistent disturbances in feeding, gut motility, bowel habits, medication exposure, oral health, and daily care routines, all of which are capable of remodeling the intestinal ecological niche. Experimental evidence also indicates that maternal microbial signals can shape fetal neurodevelopment, underscoring the potential relevance of early-life microbial environments to developmental brain vulnerability (9).

Recent reviews of the MGBA in neurodevelopmental and mental-health conditions emphasize that microbial signals may influence neuroimmune maturation, barrier regulation, microglial development, neurotransmitter metabolism, and stress responsivity (10, 11). CP is nevertheless distinct from disorders such as autism spectrum disorder or attention-deficit/hyperactivity disorder because the primary neurological insult is typically a non-progressive fetal or infant brain injury, while gut microbial remodeling may arise secondarily from neurogenic bowel dysfunction, dysphagia, tube or texture-modified feeding, reduced mobility, constipation, antiepileptic exposure, oral inflammation, and caregiver-dependent routines (12–14).

In CP, the MGBA may be clinically important at three levels. First, the primary brain injury can alter autonomic regulation, swallowing, physical activity, and bowel motility, thereby modifying intestinal transit and nutrient availability. Second, chronic constipation, liquid or texture-modified diets, malnutrition, antiepileptic drugs, antibiotics, recurrent infections, and reduced mobility may further promote microbial dysbiosis (12–14). Third, a dysbiotic microbiota and its metabolites may feed back to the host through barrier disruption, systemic inflammation, altered neurotransmitter precursor metabolism, vagal signaling, and microglial activation (15–17).

The current literature on the MGBA in CP remains fragmented. Existing studies have mainly focused on constipation, diet, epilepsy-related microbiome changes, or general gut-brain mechanisms, whereas few reviews have integrated these findings into a CP-specific framework that considers early non-progressive brain injury, neurogenic bowel dysfunction, feeding difficulty, oral-gut inflammation, medication exposure, immune-metabolic signaling, and care-environment factors together. Therefore, the knowledge gap addressed by this review is not whether the gut microbiome is broadly relevant to neurodevelopment, but how MGBA-related mechanisms may be specifically interpreted in the clinical context of CP. This review aims to fill this gap by synthesizing CP-specific clinical evidence, integrating it with mechanistic findings from broader neurodevelopmental and gut-brain research, and clarifying which MGBA pathways may represent plausible biomarkers, modifiable contributors to symptom burden, or future therapeutic targets. In doing so, the review seeks to distinguish established evidence from biologically plausible hypotheses and to provide a structured framework for future mechanistic and intervention studies in CP.

## 2 Clinical basis for gut microbial dysregulation in CP

Children with CP are particularly vulnerable to gastrointestinal and nutritional complications. Dysphagia, impaired oral motor control, delayed gastric emptying, gastroesophageal reflux, chronic constipation, low fluid intake, reduced dietary fiber, and protein-energy or micronutrient deficiencies are frequently reported (12–14). Constipation is especially common, with reported prevalence varying widely according to diagnostic criteria, motor severity, feeding route, and study setting. Epilepsy is also prevalent in CP, and seizure burden, antiepileptic medications, sleep disruption, and feeding difficulty may compound gastrointestinal dysfunction (4, 5). These clinical features create a plausible biological substrate for MGBA disturbance.

A distinctive feature of CP is that brain injury may precede and drive changes at the gut end of the axis. Injury involving cortical, subcortical, brainstem, and autonomic networks can interfere with swallowing coordination, gastrointestinal motility, secretion, visceral sensation, pelvic floor coordination, and behavioral routines around feeding and toileting. Abnormal muscle tone, limited mobility, prolonged sitting, dehydration, and difficulty with defecation training can further aggravate stool retention and intestinal transit delay (12–14). Therefore, gut dysbiosis in CP should not be viewed as an isolated primary event; it is more likely to emerge from a complex interaction between neurogenic bowel dysfunction, diet, medication, and care environment.

The oral-gut inflammatory axis may have particular relevance in CP. Oropharyngeal motor dysfunction, drooling, reduced chewing efficiency, dependence on caregivers for hygiene, high-carbohydrate feeding patterns, and impaired dental care increase the risk of dental caries, gingivitis, and oral microbial imbalance. Oral inflammation may contribute to downstream intestinal dysbiosis through continual swallowing of inflammatory and microbial products, and may be associated with constipation, inflammatory markers, and reduced quality of life (18). This suggests that MGBA assessment in CP should move beyond fecal microbiome profiling and incorporate oral microbial ecology, dental status, and feeding mechanics.

## 3 Mechanistic links between the MGBA and CP

The key studies supporting the mechanistic interpretation of the MGBA in CP are summarized in Table 1. Evidence is interpreted in three tiers throughout this section: (i) CP-specific human evidence, which is currently limited to observational microbiome/metabolome studies and constipation-oriented interventions; (ii) non-CP human evidence, which supports general MGBA plausibility but is indirect for CP; and (iii) animal or *in vitro* evidence, which can establish mechanism under experimental conditions but cannot demonstrate that the same pathway is causal in children with CP. Statements below therefore distinguish observed CP associations from extrapolated mechanisms.

**TABLE 1:** Research supporting the mechanistic link between the microbiota–gut–brain axis and cerebral palsy.

Evidence level	References	Brief description of the study	Mechanistic implication addressed	Evidence strength and study quality for CP interpretation
Animal/preclinical mechanistic studies	Braniste et al. (19)	Using germ-free and pathogen-free mice, the study showed that intestinal microbiota influenced blood-brain barrier permeability and tight-junction protein expression.	Barrier regulation: gut microbiota-BBB communication and neuroimmune interface.	Moderate preclinical mechanistic evidence; indirect for CP; no human CP validation.
Animal/preclinical mechanistic studies	Needham et al. (20)	A gut-derived metabolite was shown in mice to enter the brain and alter brain activity, myelination-related biology, and anxiety-like behavior.	Microbial metabolites: metabolite-mediated brain activity, myelination, and behavioral regulation.	Moderate preclinical evidence; indirect for CP; single experimental model.
Animal/preclinical mechanistic studies	Erny et al. (21)	Germ-free or microbiota-disrupted mice displayed abnormal microglial maturation and function, which could be partly normalized by microbial recolonization or SCFA-related signals.	Immune-microglial axis: microbiota/SCFA control of microglial maturation and neuroimmune homeostasis.	Moderate preclinical evidence; indirect for CP; germ-free/antibiotic models.
Animal/preclinical mechanistic studies	Yano et al. (22)	The study demonstrated that specific gut bacteria can regulate host serotonin biosynthesis through effects on enterochromaffin-cell metabolism.	Tryptophan-serotonin pathway: microbial regulation of neurotransmitter-related metabolism.	Moderate preclinical evidence; indirect for CP; mechanistic mouse study.
Animal/preclinical mechanistic studies	Sudo et al. (23)	Postnatal microbial colonization in mice programmed hypothalamic-pituitary-adrenal stress responses.	Neuroendocrine signaling: microbiota-HPA-axis programming and stress responsiveness.	Moderate preclinical evidence; indirect for CP; developmental timing limits translation.
Animal/preclinical mechanistic studies	Bravo et al. (24)	*Lactobacillus rhamnosus* exposure altered central GABA-receptor expression and stress-related behavior in mice, and these effects depended on an intact vagus nerve.	Neural pathway: vagus-dependent gut-brain signaling and inhibitory neurotransmission.	Moderate preclinical evidence; indirect for CP; strain- and model-specific.
Human observational studies	Huang et al. (15)	In children with CP and epilepsy, different dietary structures were associated with different gut microbial profiles and gastrointestinal dysfunction, especially constipation-related features.	Diet-motility-microbiota link: liquid/low-fiber diet, SCFA-producing taxa, and constipation-related dysbiosis.	Low CP-specific evidence; observational and confounded by epilepsy, diet and feeding mode.
Human observational studies	Peng et al. (16)	Multi-omics analysis in children with CP and epilepsy identified gut microbiome and metabolomic remodeling related to immune and neurodevelopmental pathways.	Microbial-metabolic remodeling: links among dysbiosis, immune signaling, and neurodevelopment-related metabolism.	Low-to-moderate CP-specific evidence; multi-omics strength but cross-sectional and non-causal.
Intervention studies	García-Contreras et al. (25)	A double-blind randomized placebo-controlled trial tested *Lactobacillus reuteri* DSM 17938 and/or agave inulin in children with CP and chronic constipation and reported improvements in selected stool-related outcomes.	Microbiome-targeted bowel modulation: probiotic/prebiotic effects on constipation phenotype and intestinal milieu.	Moderate evidence for constipation outcomes; randomized design; not evidence of neurological benefit.
Intervention studies	Huang et al. (26)	A clinical trial using dietary fiber combined with probiotics in children with CP and functional constipation reported improved constipation and gut microbiota restructuring.	Substrate-based microbial modulation: fiber/probiotics, protective bacteria, and functional constipation improvement.	Low-to-moderate evidence for constipation and microbial change; limited sample and follow-up.

CP, cerebral palsy; MGBA, microbiota-gut-brain axis; BBB, blood-brain barrier; SCFAs, short-chain fatty acids; HPA, hypothalamic-pituitary-adrenal.

### 3.1 Intestinal barrier disruption and amplification of systemic inflammation

A healthy intestinal microbiota supports epithelial integrity by promoting mucus production, maintaining tight junction proteins, competing with pathogens, and generating SCFAs. In CP, chronic constipation, limited dietary diversity, liquid diets, and low fiber intake may reduce the abundance of butyrate-producing and fiber-fermenting bacteria while promoting opportunistic taxa (15–17). When the intestinal barrier is compromised, bacterial products such as lipopolysaccharide can enter the circulation, activate innate immunity, and increase systemic cytokine signaling. These signals may influence the central nervous system through endothelial activation, blood-brain barrier modulation, and immune cell trafficking (27–29). In a child with pre-existing developmental brain injury, low-grade systemic inflammation may increase the vulnerability of neural networks and worsen symptom burden.

The blood-brain barrier is another key interface in MGBA signaling. Animal studies have shown that microbial colonization and microbial metabolites can influence blood-brain barrier permeability and immune tone, while gut inflammation and host inflammasome activity can amplify neuroinflammatory signals (19, 30, 31). Although direct evidence in CP remains limited, the combination of early brain injury, recurrent systemic inflammation, constipation, and nutritional compromise provides a biologically coherent context in which peripheral immune activity could interact with central vulnerability. At the same time, barrier signaling should be considered together with the broader gut-brain-immune interface, because neuroinflammatory amplification may depend on both epithelial and blood-brain barrier regulation rather than on a single anatomical compartment (32, 33).

Evidence status—CP-specific studies associate diet, constipation and selected taxa with gastrointestinal phenotypes, whereas epithelial permeability, lipopolysaccharide translocation and central inflammatory amplification are inferred mainly from general MGBA and experimental studies.

### 3.2 Short-chain fatty acid depletion and dysregulated glial modulation

Beyond classical SCFA signaling, other gut-derived metabolites may also modulate neural activity and behavior. Experimental evidence that a gut-derived metabolite can alter brain activity and anxiety-like behavior supports the broader concept that microbial metabolites may serve as an intermediate layer between intestinal ecology and CP-related emotional, sensory, pain, and adaptive-behavior phenotypes (20).

Short-chain fatty acids, principally acetate, propionate, and butyrate, are important metabolic mediators of gut-brain communication. They serve as energy substrates for colonocytes, enhance epithelial barrier function, modulate immune cell differentiation, and regulate microglial maturation and neuroimmune homeostasis through receptor-mediated and epigenetic mechanisms (21, 34, 35). Germ-free and antibiotic-treated animal models demonstrate abnormal microglial development and reactivity, which can be partly rescued by microbial recolonization or SCFA supplementation (21, 34). CP-specific studies have not yet established a direct causal chain between SCFA deficiency and neuroinflammation; nevertheless, reduced abundance of taxa such as *Roseburia*, *Faecalibacterium*, *Ruminococcus*, and related fiber-fermenting organisms in selected CP cohorts suggests a potentially important metabolic gap (15, 16).

Evidence status — selected CP cohorts report reduced abundance of potential SCFA-producing taxa, but a causal SCFA–microglia pathway has not been demonstrated in CP; the mechanistic evidence is primarily preclinical and non-CP.

### 3.3 Immune-microglial axis and persistent neuroinflammation

The immune-microglial axis is central to the MGBA concept. Gut microbial molecules, SCFAs, secondary bile acids, and mucosal cytokines can shape the balance between regulatory and pro-inflammatory immune programs, including Treg and Th17-related responses (28, 29). Microglia are essential for synaptic pruning, neurogenesis, oligodendrocyte support, and circuit refinement during development. If microglia remain chronically primed toward a pro-inflammatory phenotype, they may contribute to maladaptive network remodeling and impaired repair after early injury (21, 36). Because CP often follows hypoxic-ischemic injury, infection, prematurity, or perinatal inflammation, dysbiotic gut-derived immune stimulation may act as a post-injury second hit that maintains neuroinflammatory pressure. This link is consistent with broader models in which the gut microbiome communicates with the central nervous system through immune and neural pathways (37, 38). Recent reviews also highlight microglia and gut-immune-brain circuitry as mechanistic nodes connecting peripheral microbial signals to neuroinflammation (39, 40).

Recent neurodevelopmental MGBA literature further supports an immune-metabolic model in which dysbiosis may increase microbial product exposure, cytokine tone, SCFA and bile-acid imbalance, tryptophan-kynurenine flux, and microglial activation (11, 28, 29). In CP, these mechanisms should be interpreted as modifiers of vulnerability after early brain injury rather than as direct evidence that dysbiosis causes CP. A more precise hypothesis is that constipation, diet restriction, medication exposure, recurrent infection, and reduced mobility may sustain peripheral inflammatory signals that interact with already vulnerable developing neural circuits.

Evidence status—microglial activation by gut-derived signals remains an extrapolated mechanism in CP. CP-specific studies have not directly linked microbial features to microglial state, whereas animal and broader neuroimmune studies support biological plausibility.

### 3.4 Tryptophan-kynurenine pathway and neurotransmitter imbalance

Tryptophan metabolism provides another mechanistic bridge. The microbiota can influence the partitioning of tryptophan toward serotonin, indole derivatives, or the kynurenine pathway (22, 41). Excessive kynurenine pathway activation is frequently associated with inflammation and can generate neuroactive metabolites with excitatory, oxidative, or immunomodulatory effects. In contrast, certain microbial indoles support epithelial and immune homeostasis (41, 42). In CP, constipation, chronic inflammation, epilepsy, disrupted sleep, pain, and altered diet may promote shifts in tryptophan metabolism that influence mood, visceral sensitivity, nociception, sleep, and neural excitability (4, 16). Because the gut microbiota can also function as an endocrine-like organ, these metabolic shifts should be interpreted within a wider gut-tryptophan-brain and neurological-disease framework rather than as an isolated serotonin or kynurenine abnormality (43–45). This immune-metabolic framing also links tryptophan diversion toward kynurenine metabolites with microglial activation and altered excitatory-inhibitory balance in broader neurodevelopmental MGBA research (11, 41).

Evidence status—CP-specific metabolomic observations support association, not pathway causality; microbial control of serotonin, indole and kynurenine metabolism is derived mainly from experimental and non-CP evidence.

### 3.5 Vagus nerve, enteric nervous system, and HPA-axis dysregulation

Neural and endocrine communication pathways also deserve attention. Microbial metabolites and enteroendocrine signals may activate vagal afferents, enteric neurons, and brainstem circuits, whereas chronic stress and hypothalamic-pituitary-adrenal (HPA) axis activation can alter gut permeability, motility, and microbial composition (23, 24, 46). Children with CP may experience chronic pain, sleep disruption, frequent medical procedures, caregiver-mediated stress, and limited autonomy, all of which may interact with the HPA axis and gut physiology. Although direct human evidence in CP is sparse, vagal, enteric, and stress-related pathways offer a plausible explanation for the co-occurrence of gastrointestinal symptoms, sleep disturbance, pain, and behavioral dysregulation (47–49).

**FIGURE 1:**
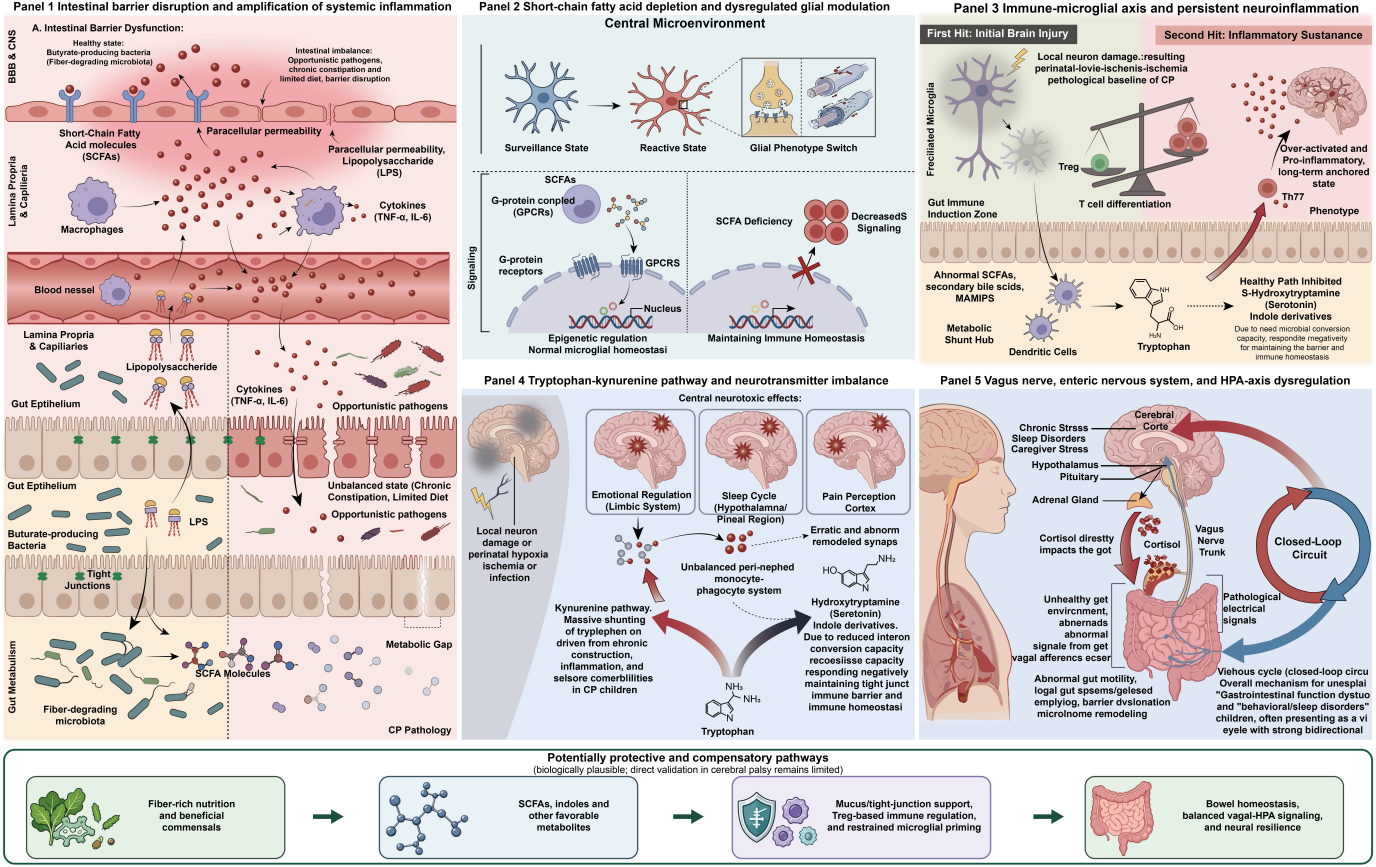
Conceptual framework of the microbiota-gut-brain axis (MGBA) in cerebral palsy. Primary developmental brain injury may impair feeding, autonomic regulation, mobility, and bowel function, thereby promoting constipation and microbiome remodeling. Dysbiosis may then feedback through barrier-immune, metabolic, neural, and endocrine pathways. The Figure summarizes interfaces that may become biomarkers or therapeutic targets in future studies. The lower panel balances the pathogenic model by showing protective and compensatory routes through which beneficial fiber-fermenting microbiota, SCFAs and indole derivatives may reinforce tight junctions, support Treg-associated anti-inflammatory signaling, maintain homeostatic microglia, and stabilize vagal–HPA regulation. These protective routes are mechanistically plausible and are not yet validated as causal therapeutic pathways in CP.

Evidence status — vagal and HPA-axis mechanisms are supported mainly by animal and general MGBA studies. Direct evidence that these pathways mediate CP symptoms in humans is currently absent. The major MGBA nodes, possible CP-related alterations, and corresponding clinical or research implications are summarized in Table 2. The integrated CP-specific MGBA framework, including pathogenic feedback loops and potentially protective or compensatory routes, is shown in Figure 1.

**TABLE 2:** Major MGBA nodes relevant to cerebral palsy.

MGBA node	Potential alteration in CP	Pathophysiological implication	Clinical/research implication
Gut motility and feeding	Dysphagia, delayed transit, constipation, liquid or low-fiber diet	Alters microbial substrate availability and ecological stability	Assess feeding route, texture, hydration, stool burden, and diet before microbiome interpretation
Barrier and immune interface	Epithelial stress, increased permeability, microbial product translocation	Promotes systemic cytokine signaling and may interact with blood-brain barrier responses	Measure inflammatory markers, barrier markers, and constipation severity alongside microbiota
Microbial metabolites	Reduced SCFA-producing taxa and altered tryptophan metabolism	May influence epithelial integrity, immune tone, microglia, pain, sleep, and excitability	Integrate metabolomics rather than relying on 16S taxonomy alone
Neural/endocrine routes	Vagal, enteric nervous system, and HPA-axis dysregulation under chronic stress and pain	Links bowel dysfunction with sleep, behavior, and visceral sensitivity	Include sleep, pain, caregiver stress, and autonomic measures in trials
Oral-gut ecology	Caries, gingivitis, oral dysbiosis, swallowed inflammatory load	May contribute to downstream gut inflammation and dysbiosis	Add dental and oral microbiome assessment to CP microecology studies

MGBA, microbiota-gut-brain axis; CP, cerebral palsy; SCFAs, short-chain fatty acids; HPA, hypothalamic-pituitary-adrenal.

## 4 Current clinical evidence for MGBA abnormalities in CP

Clinical microbiome research in CP is still emerging, but available studies suggest that microbial dysbiosis may relate to both gastrointestinal and neurological phenotypes. Peng et al. compared the gut microbiota and metabolomic profiles of children with CP and epilepsy with controls and reported microbial imbalance, functional pathway changes, and metabolic alterations involving immune and neurodevelopmental pathways (16). Subsequent work has suggested that microbiome changes may be more pronounced in children with CP and epilepsy, implying that comorbidity can amplify MGBA disturbance (17). These observations challenge a purely bowel-centered explanation and support the possibility that microbial features in CP reflect an intersection of gut function, neurodevelopment, and immune regulation.

Dietary structure appears to be a major modifiable driver. In children with CP and epilepsy, liquid diets have been associated with increased abundance of taxa such as *Collinsella*, *Alistipes*, and *Eggerthella* and with reduced abundance of several bacteria involved in fiber fermentation and SCFA production, including *Lachnoclostridium*, *Dorea*, *Ruminococcus*, *Faecalibacterium*, *Roseburia*, and *Lactobacillus* (15). These alterations were linked with constipation and nutritional phenotypes, indicating that diet is not merely a background covariate but a major determinant of microbial ecology in CP. For children with severe dysphagia, tube feeding, or long-term semi-liquid diets, the microbial and metabolic consequences of nutritional formulas require more systematic evaluation.

Care patterns and psychosocial context may also influence the MGBA. A recent study reported that different care modes altered gut microbiota composition and function in children with CP, with family-centered care associated with more favorable microbial features (50). This finding broadens the scope of CP microbiome research by suggesting that feeding behavior, daily activity, parent-child interaction, stress level, sleep routine, and rehabilitation adherence may all be biologically reflected through microbial ecology. The MGBA should therefore be considered an integrated care-environment axis rather than a purely microbial phenomenon.

Evidence from other neurodevelopmental and neurodegenerative conditions supports the biological plausibility of microbiome-driven modulation of brain-related phenotypes, although such findings cannot be directly extrapolated to CP. In autism spectrum disorder models, human microbiota transfer can influence behavioral abnormalities, whereas in Parkinson disease models the microbiota can regulate motor deficits and neuroinflammation (51, 52). These studies provide proof-of-concept that gut microbial signals can affect the nervous system, but CP-specific causal studies are needed before therapeutic claims can be made.

Epilepsy and its treatment remain important confounders and potential effect modifiers. Epilepsy is common in CP, and antiepileptic drugs, seizure burden, altered sleep, feeding route, and recurrent infections may each influence the gut microbiome (4). Existing studies indicate stronger microbial alteration in children with CP and epilepsy, but the independent contributions of CP itself, epilepsy, antiepileptic medication, and diet remain difficult to separate (16, 17). Future studies must adopt careful phenotyping, medication adjustment, and longitudinal sampling to improve causal inference.

### 4.1 Critical appraisal of CP-specific microbiome evidence

The principal strength of the CP literature is its direct clinical relevance: available studies link microbial variation to feeding structure, constipation, epilepsy-related phenotypes, and metabolic remodeling (15–17). Care context has also been associated with microbial composition and function (50). However, the reported taxonomic patterns are not sufficiently concordant to define a reproducible CP-specific microbiome signature. Differences in inclusion criteria, comparator selection, bowel phenotype, dietary exposure, medication burden and analytical workflow can change both the direction and magnitude of taxon-level associations. Consequently, depletion of individual genera or enrichment of opportunistic taxa should be interpreted as cohort-specific signals rather than as universal biomarkers.

Intervention evidence is somewhat stronger for bowel outcomes than for neurodevelopmental mechanisms. Randomized or prospective studies of inulin, probiotics and dietary fiber support improvements in selected constipation measures and microbial indices (25, 26), but they are small, short-term and heterogeneous in formulation and outcomes. They do not establish that modifying the microbiome improves motor, cognitive, seizure or long-term developmental outcomes. Thus, the most defensible current conclusion is that diet, bowel dysfunction and care exposures are associated with microbial remodeling in CP, while disease-specific taxa, causal neurological pathways and durable neurodevelopmental benefit remain unresolved.

### 4.2 CP-specific heterogeneity and sources of cross-study discordance

Cerebral palsy is not a biologically uniform exposure. Epilepsy and seizure burden add further clinical heterogeneity (4, 5). Gross Motor Function Classification System level and motor subtype influence mobility, energy requirements and intestinal transit; oral versus tube or texture-modified feeding changes substrate delivery; malnutrition and micronutrient deficiency alter host–microbe metabolism; and constipation changes transit time and stool water content (12–14). Antiepileptic drug exposure and diet may also contribute to variation in CP-associated microbiome profiles (15–17). Oral health and family-care environment are additional modifiers of microbial composition (18, 50). Prematurity, age, geography, lesion pattern, antibiotic exposure and recurrent infections should therefore be systematically recorded and considered in analysis. Because these variables are often unevenly measured or distributed across small cohorts, an apparent CP-associated taxon may instead reflect feeding severity, bowel transit, comorbidity or treatment. Future studies should therefore use harmonized phenotyping, prespecified stratification and matched or adjusted analyses rather than pooling clinically dissimilar children.

## 5 Therapeutic strategies from an MGBA perspective

Microbiota-gut-brain axis-directed therapy in CP should begin with foundational gastrointestinal and nutritional management. Children with dysphagia, malnutrition, chronic constipation, or feeding difficulty should undergo structured nutritional assessment, growth monitoring, micronutrient evaluation, feeding-route optimization, hydration review, dietary fiber assessment, and standardized constipation treatment (12–14). If fecal impaction, neurogenic bowel dysfunction, low fluid intake, or major dietary imbalance remain unaddressed, isolated probiotic supplementation is unlikely to produce durable ecological restoration. Multidisciplinary management involving neurology, rehabilitation, gastroenterology, nutrition, dentistry, nursing, and family caregivers is therefore essential.

The most direct MGBA-targeted clinical evidence in CP comes from constipation-oriented trials. A double-blind randomized placebo-controlled trial reported that *Lactobacillus reuteri* DSM 17938 and/or agave inulin improved selected defecation outcomes in children with CP and chronic constipation, including painful defecation, stool retention, and rectal stool burden (25). Another study suggested that dietary fiber combined with probiotics could improve functional constipation in CP and was associated with microbial restructuring (26). These findings indicate that probiotics and prebiotics may provide benefits beyond laxation by influencing microbial metabolism, intestinal barrier function, and inflammatory tone. Other CP constipation studies, including comparisons of therapeutic approaches and body-based interventions such as massage or Tui Na, further indicate that bowel-directed care can be clinically relevant even when microbiome endpoints are not the primary focus (53–55).

However, current probiotic, prebiotic, and synbiotic evidence remains insufficient for broad neurodevelopmental claims. Most studies are small, short-term, and heterogeneous in strain selection, dose, treatment duration, concomitant diet, and outcome assessment. Outcomes usually emphasize stool frequency or consistency rather than sleep, behavior, cognition, epilepsy control, pain, or long-term rehabilitation response (25, 26). Live biotherapeutic products also require careful safety consideration in children with severe dysphagia, gastrostomy, immune vulnerability, recurrent aspiration, or frequent hospitalization (12–14). At present, these agents are best regarded as adjuncts within comprehensive care rather than substitutes for standard bowel and nutritional therapy. Broader prebiotic, probiotic, and dietary modulation literature supports MGBA biological plausibility, but it also reinforces the need to distinguish bowel-function improvement from proven neurodevelopmental benefit (56, 57).

Evidence from inflammatory and immune-mediated disorders also supports a broader rationale for microbiome-targeted therapies, including dietary substrate modification, probiotics, prebiotics, and strategies aimed at restoring mucosal immune balance and epithelial repair (58). For CP, this broader literature strengthens biological plausibility but does not replace the need for CP-specific pediatric trials that control for feeding route, constipation severity, medication exposure, and baseline nutritional status.

Dietary fiber and diet reconstruction may be among the most scalable interventions. Fiber increases stool bulk, supports colonic motility, and provides substrate for SCFA-producing bacteria (15, 26). For children dependent on liquid or semi-liquid diets, the challenge is to increase fermentable substrates while maintaining swallowing safety, caloric adequacy, gastrointestinal tolerance, and caregiver feasibility. Compared with single-strain supplementation, sustained dietary optimization may produce more stable microbial restructuring because it modifies the ecological substrate that maintains microbial communities (12–14).

Oral health management is a potentially underrecognized MGBA intervention. Improving oral hygiene, treating periodontal inflammation, reducing cariogenic feeding residues, and integrating swallowing and dental care may reduce inflammatory microbial load and downstream gut exposure (18). Combined profiling of oral microbiota, fecal microbiota, inflammatory markers, and gastrointestinal symptoms may help define clinically meaningful microecological subtypes of CP.

Family-centered care and rehabilitation ecology also warrant consideration. The upstream determinants of the MGBA include feeding behavior, activity, sleep, stress, daily routines, caregiver training, and psychosocial support. Family-centered care has been associated with favorable gut microbial functional features in CP (50). Therefore, MGBA-oriented CP management should be conceptualized as a nutrition-behavior-family-microbiome strategy, not as a single supplement-based treatment.

Postbiotics, specific microbial metabolites, fecal microbiota transplantation (FMT), and precision nutrition represent future directions. Postbiotics may offer greater stability and safety than live organisms, FMT may theoretically correct severe dysbiosis, and precision nutrition could tailor substrate delivery to feeding route, constipation phenotype, seizure comorbidity, and microbial-metabolic profile (29, 59). Nevertheless, these approaches lack sufficient evidence in CP, particularly regarding neurodevelopmental benefit, long-term safety, drug-microbiome interactions, and ethical implementation in children. For now, they should remain within well-designed research frameworks.

Fecal microbiota transplantation requires particular caution in pediatric CP. Beyond uncertain efficacy for neurodevelopmental outcomes, the procedure raises ethical, regulatory, and safety issues, including assent and parental consent, donor selection and repeated screening, transmission of pathogens or antimicrobial-resistance genes, product standardization, engraftment monitoring, long-term follow-up, and the heightened vulnerability of children with dysphagia, gastrostomy dependence, recurrent aspiration, malnutrition, immune compromise, or frequent hospitalization (60–62). Therefore, FMT should not be presented as routine MGBA therapy for CP and should be restricted to ethically approved, regulatorily compliant protocols with predefined safety stopping rules. The potential MGBA-oriented intervention layers in CP are summarized in Table 3.

**TABLE 3:** Potential microbiota-gut-brain axis (MGBA)-oriented intervention layers in cerebral palsy (CP).

Intervention layer	Candidate strategy	Main expected effect	Evidence maturity in CP
Foundational care	Nutritional assessment, hydration, fiber review, feeding-route optimization, standard constipation protocol	Corrects dominant upstream drivers of dysbiosis and stool retention	Established clinical need; microbiome-specific evidence still developing
Microbiome modulation	Probiotics, prebiotics, synbiotics, dietary fiber	Improves stool patterns and may increase favorable fermentation	Promising but mainly small studies with bowel outcomes
Oral health	Dental care, periodontal inflammation control, caregiver hygiene training	Reduces oral inflammatory and microbial burden entering the gut	Mechanistically plausible; CP-specific integrated trials needed
Family-centered ecology	Caregiver training, feeding routines, sleep and stress management, activity support	Modifies behavioral and environmental determinants of the MGBA	Emerging evidence; requires controlled longitudinal evaluation
Precision approaches	Postbiotics, targeted metabolites, precision nutrition, FMT in research settings	Aims at functional microbial-metabolic restoration	Experimental; safety and efficacy not established for routine CP care

FMT, fecal microbiota transplantation. Precision and transplantation-based strategies should currently be limited to research settings in cerebral palsy (CP).

## 6 Limitations

### 6.1 Limitations of current evidence

The MGBA offers an integrative framework for CP, but the existing evidence base has important limitations. CP-related microbiome studies are generally small and heterogeneous. Motor severity, Gross Motor Function Classification System level, lesion type, epilepsy status, feeding route, constipation severity, antibiotic exposure, antiepileptic medication, dental status, and socioeconomic context can all influence microbial composition (15–17). Without standardized phenotyping, microbiome signatures may reflect confounding rather than disease-specific biology.

Most available studies are cross-sectional. They can identify associations, but they cannot establish whether brain injury causes dysbiosis, dysbiosis amplifies symptoms, or both processes interact over time (16, 17). Longitudinal studies beginning early in life, ideally in high-risk infants and stratified by feeding and injury characteristics, are needed to define temporal sequence and potential windows of intervention.

Many studies rely on fecal 16S rRNA sequencing and bowel outcomes. This approach provides useful taxonomic information but insufficient functional resolution. Integrated metagenomics, metatranscriptomics, metabolomics, immune profiling, oral microbiome assessment, neuroimaging, electrophysiological measures, and standardized clinical outcomes are needed to test mechanistic hypotheses (16, 18). Childhood microbiomes are also developmentally dynamic, and age, delivery mode, early feeding, geography, medication exposure, and diet can strongly affect reproducibility (6–8).

The broader MGBA research field has also been criticized for overinterpretation, heterogeneity, and insufficient causal testing. The paradigm has advanced neuroscience by emphasizing biological communication between gut microbes and the brain, but it also requires rigorous study design, transparent statistics, and avoidance of reductionist claims (63, 64). In CP, the MGBA should be framed as a modifiable contributor to symptom burden and treatment response rather than as a replacement for the established neurodevelopmental basis of the condition.

### 6.2 Methodological of limitations

Clinical confounding and technical variation are inseparable concerns in current CP microbiome research. Across human disease studies, bowel habit, diet, age, geography, medication and other host variables can generate spurious case–control associations when they are not balanced or modeled (65). This risk is amplified in CP because these exposures track with motor severity, feeding route, epilepsy and care dependence. Multicenter studies should therefore collect a core covariate set, prespecify matching or adjustment, document sampling time relative to antibiotics and bowel interventions, and analyze constipation and feeding phenotypes as potential effect modifiers rather than nuisance variables.

Most CP studies use 16S rRNA amplicon sequencing, which is economical for broad bacterial community profiling but has limited species/strain resolution, is sensitive to primer and variable-region choice, and infers function indirectly. Shotgun metagenomics can resolve genes and pathways and profile a broader range of microorganisms, but requires greater sequencing depth, host-DNA control, computational resources and curated reference databases (66). Differences in stool collection, storage, DNA extraction, amplification, library preparation, sequencing platform and bioinformatics pipeline can create batch effects comparable with or larger than biological signals (67). Cross-study pooling without technical harmonization is therefore hazardous.

Microbiome count data are compositional: an apparent increase in one taxon can arise from decreases elsewhere, and analyses based on untransformed relative abundance may generate misleading associations (68). Unequal sequencing depth, low biomass, contamination and taxon-specific extraction or amplification efficiency introduce additional bias (69). Reproducible CP research should use negative and positive controls, report read depth and filtering, account for batch and multiple testing, apply composition-aware statistics, validate findings in an independent cohort, deposit code and metadata, and follow microbiome-specific reporting guidance such as STORMS (70). These safeguards are prerequisites for moving from descriptive taxon lists to credible biomarkers.

## 7 Future directions

Future MGBA research in CP should move toward multicenter, deeply phenotyped, longitudinal cohorts. Participants should be stratified by CP subtype, motor severity, epilepsy status, feeding route, constipation phenotype, oral health, antibiotic and antiepileptic drug exposure, nutritional status, and rehabilitation context. Such stratification is essential to distinguish disease-associated microbial features from diet- or medication-driven signals.

Future studies should combine shotgun metagenomics with targeted and untargeted metabolomics, metatranscriptomics and host immune profiling, then integrate these layers using systems-biology and network approaches (71). Fecal and oral microbiota should be analyzed together with SCFAs, bile acids, tryptophan metabolites, inflammatory and epithelial-barrier markers, neuroimaging, sleep, seizure, motor and cognitive outcomes. Such functional integration can identify pathway-level biomarkers and therapeutic targets that are more transferable than isolated taxonomic associations.

Artificial-intelligence and machine-learning methods may assist patient stratification, multi-omics feature selection and prediction of treatment response (72). Their use should remain hypothesis-generating until models are externally validated across centers, calibrated for small and imbalanced pediatric cohorts, tested against clinical baselines, and interpreted with transparent feature attribution. Data leakage, overfitting and site-specific batch effects could otherwise produce apparently accurate but non-generalizable CP microbiome classifiers.

Mechanism-oriented randomized controlled trials should evaluate dietary fiber, defined probiotic or synbiotic combinations, postbiotics, targeted nutritional formulas, oral-health interventions, and family-centered care bundles. Trials should include not only bowel outcomes but also nutrition, sleep, pain, epilepsy control, behavior, caregiver burden, and rehabilitation responsiveness (25, 26). Follow-up should be long enough to assess durability and relapse after intervention withdrawal.

Translational models are needed to improve causal inference. Animal and cellular models can test whether CP-relevant dysbiosis, microbial metabolites, or inflammatory signatures alter gut motility, microglial activation, myelination, motor behavior, or seizure susceptibility. Lessons from other neurodevelopmental models, where microbial manipulation can modify behavioral and physiological abnormalities, provide a useful framework for designing CP-specific mechanistic studies (73). Ultimately, the field should aim to connect mechanism, biomarker, intervention, and patient-centered outcome in a coherent translational pipeline.

## 8 Conclusion

The MGBA provides a cross-system framework for understanding why children with CP frequently experience intertwined neurological, gastrointestinal, immune, nutritional, and behavioral symptoms. Current evidence suggests that diet, constipation, epilepsy, medication exposure, oral inflammation, and care environment can shape gut microbial ecology in CP (4, 5). Dysbiotic microbial communities and their metabolites may contribute to symptom persistence through barrier disruption, systemic inflammation, immune imbalance, altered tryptophan metabolism, vagal and enteric signaling, HPA-axis dysregulation, and microglial priming (27–29).

At present, MGBA-oriented interventions such as dietary fiber, probiotics, prebiotics, synbiotics, oral health management, nutritional optimization, constipation protocols, and family-centered care appear promising mainly for bowel function and short-term microbial modulation (26, 50). Evidence remains insufficient to establish standardized neurodevelopmental treatment protocols. The MGBA should therefore not be interpreted as shifting CP etiology to the gut; rather, it identifies a potentially modifiable biological layer superimposed on early brain injury. Incorporating this layer into life-course CP management may support more precise, integrated, and personalized care.

Thus, the present evidence remains preliminary: current interventions mainly support gastrointestinal symptom relief and short-term microbial or inflammatory modulation, whereas evidence that MGBA-targeted strategies improve neurological or developmental outcomes in CP is still insufficient. Clinical translation now depends on four priorities: multicenter longitudinal cohorts with harmonized CP phenotyping and sample processing; direct measurement and experimental validation of microbial functions rather than taxonomic inference alone; adequately powered, preregistered trials using standardized interventions, safety monitoring and clinically meaningful neurological as well as gastrointestinal outcomes; and independent external validation of biomarkers across age, geography, feeding route and comorbidity strata. Until these requirements are met, MGBA markers and therapies should remain adjunctive research tools rather than determinants of routine CP diagnosis or neurorehabilitation.
